# Migrants Living in the United Kingdom and Their Perceptions of Participation in Health Research: A Mixed‐Methods Study

**DOI:** 10.1111/hex.70337

**Published:** 2025-06-30

**Authors:** Mayuri Gogoi, Rebecca F. Baggaley, Luisa Silva, Zainab Lal, Holly Reilly, Vishant Modhwadia, Daniel Pan, Paul Bird, Laura Nellums, Manish Pareek

**Affiliations:** ^1^ Department of Respiratory Sciences University of Leicester Leicester UK; ^2^ Development Centre for Population Health University of Leicester Leicester UK; ^3^ Institute of Health Informatics, University College London London UK; ^4^ Department of Infectious Diseases and HIV Medicine University Hospitals of Leicester NHS Trust Leicester UK; ^5^ Li Ka Shing Centre for Health Information and Discovery, Oxford Big Data Institute University of Oxford Oxford UK; ^6^ WHO Collaborating Centre for Infectious Disease Epidemiology and Control, School of Public Health, Li Ka Shing Faculty of Medicine University of Hong Kong Hong Kong China; ^7^ Department of Clinical Microbiology University Hospitals of Leicester NHS Trust Leicester UK; ^8^ NIHR Applied Research Collaboration East Midlands (ARC EM) University of Leicester Leicester UK; ^9^ College of Population Health, Health Sciences Center University of New Mexico Albuquerque New Mexico USA; ^10^ NIHR Leicester Biomedical Research Centre (BRC) University of Leicester Leicester UK

**Keywords:** capabilities (C), COM‐B framework, health research, migrants, motivations (M), opportunities (O), research participation

## Abstract

**Background:**

Migrants' participation in health research is essential to give voice to their needs and inform evidence‐based practice. We conducted a mixed‐methods study with migrants living in Leicester, United Kingdom, to understand their perceptions of participation in health research and factors influencing participation.

**Methods:**

Our study included a questionnaire and focus groups with migrants. Interviews and focus groups were also conducted with key informants. The study was carried out at two sites in Leicester. Questionnaire data were analysed descriptively in R. The COM‐B framework was used to thematically analyse interview and focus group transcripts. Workshops with public members of migrant origin helped with data interpretation and analysis.

**Results:**

119 questionnaires and 4 focus groups (*n* = 28) were completed with migrants. Seven interviews and one focus group (*n* = 7) were conducted with key informants. Questionnaire respondents originated from 34 different countries, with a significant proportion (25%) identifying themselves as asylum seekers/refugees. Migrants in the focus groups were from 16 different countries and were mainly asylum seekers/refugees (*n* = 18). The three components of the COM‐B model (Capability, Opportunity and Motivation) were identified as the main themes, and descriptive statistics from the questionnaire data have been used to supplement the 16 sub‐themes. Individual capabilities encompassing awareness and perception of research, language abilities and skills in the use of technology significantly influenced participation. Simultaneously, the presence or absence of opportunities such as costs, competing needs and priorities, healthcare access and experiences in the United Kingdom, language barriers, opportunities for learning and taking part, precarious living conditions and socio‐cultural norms and perceptions about health were found to be important for research participation. Motivations to take part in research included trust, context of the research, need‐based research, altruism, desire to be heard and receiving incentives.

**Conclusion:**

Our study contributes to the limited evidence base exploring migrants' participation in health research. Our findings, grounded in the COM‐B model, exhibit how migrants' motivations, influenced by a host of individual capabilities and environmental and social opportunities, can influence motivation and impact research participation behaviour. These findings may support the design of accessible, inclusive, equitable and impactful health research involving underserved groups.

**Patient or Public Contribution:**

Patient and Public Involvement and Engagement (PPIE) in the project was obtained through the EMBRACE (East Midlands Migrant Research Advisory Collaborative) group, which was created as a migrant specific advisory group in 2019. We recruited new migrant members into the group and involved them in the interpretation of the study results. We organised two workshops with the group, and in the first workshop, held in February 2024, nine members took part to review the preliminary results and offer insights in contextualising and interpreting the data. The research team took into consideration the feedback received at the workshop and integrated it into the analysis. The final analysis was presented to the group again in September 2024, and the discussions held at that workshop were instrumental in shaping this manuscript.

## 1 Introduction

Migration to the United Kingdom has increased considerably in recent years. Latest figures published in 2022 show that an estimated 16% of the United Kingdom's population is comprised of non‐UK‐born residents, a 34% increase from 2011 [1]. Migration is an important determinant of health, which creates conditions and contexts that could lead to inequalities in health between migrant and host populations and even among different types of migrant groups [2]. For instance, although most newly arrived migrants to the United Kingdom report better health compared to the local population, this advantage seems to diminish over time [3]. Furthermore, certain types of migrants, such as those in low‐skilled jobs, asylum seekers and undocumented migrants, are more likely to have poorer health and face barriers to healthcare access [3, 4]. The approach to addressing migrant health concerns in the United Kingdom has largely been emergency‐driven and surveillance‐oriented, focusing mostly on infectious diseases and mental health [5]. There is, however, a growing need to focus on the holistic health of migrants, including preventative health, and to make services accessible and available for them. To achieve this, migrants' participation in health research is essential to give voice to their needs and inform evidence‐based practice [6]. Research participation has often been used coterminously with ‘research engagement’ or ‘research involvement’, but while these two terms indicate active involvement of lay members in the design, conduct and/or dissemination of research, participation refers to people taking part in a study or trial with informed consent.

Research participation, however, comes with its own set of challenges, with previous studies reporting low rates of participation from certain socially disadvantaged groups, including ethnic minorities and migrants [7, 8, 9]. For instance, diversity data published by the National Institute for Health and Care Research (NIHR), United Kingdom, from 148 randomised control trials (RCTs) initiated in a 10‐year period (2007–17) found that only 4% participants were Black and 5% identified as Asian, compared to 86% participants who identified as White [10]. While research on participation of migrants is limited, studies on under‐representation of ethnic minorities in research have highlighted several inhibiting factors, which include costs and lack of proper funding, faults in research design, cultural and linguistic barriers, lack of awareness and understanding among research teams about recruiting underserved populations, experiences of racism, mistrust and stigma [11, 12, 13, 14, 15, 16]. Although most of these studies have reported on ethnic minorities, the findings may indicate factors influencing the participation of migrant groups in research, considering the overlapping categories. Nevertheless, special attention is needed to understand research participation among migrants, given the potential influence of past experiences in home countries, migration journey and migration status on health and access to healthcare, which can thereby impact participation in health research.

We, therefore, undertook the IMMERSE (Innovative Methods of Migrant Engagement in health Research to develop Sustainable ways of mutual learning and co‐design in the East Midlands) project to conduct research and engagement activities with migrant groups living in Leicester, United Kingdom. We present data from the mixed‐methods study conducted as part of the project, and the objectives of the study were to: (a) understand migrants' knowledge and perception about participation in health research and (b) delineate the personal and social contexts within which the behaviour of research participation among migrants takes places (or is inhibited).

## 2 Theoretical Framework

**Figure 1:**
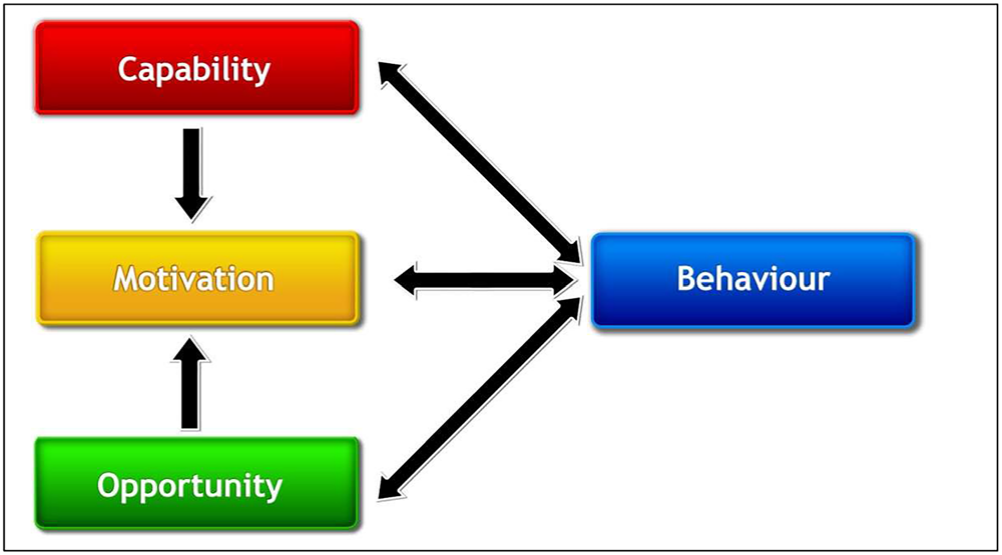
COM‐B Framework. *Source:* Michie et al. (2011).

Following Johnson et al. [17], who used the COM‐B framework to understand research participation behaviour among pharmacists, we applied this model to understand the determinants influencing research participation behaviour among migrants. This comprehensive model was conceptualised primarily to aid intervention development for behaviour change and posits that for any behaviour (B) to take place there has to be interaction between three components, Capability (C), Opportunity (O) and Motivation (M), and these in turn are influenced by the behaviour itself [18] (Figure 1). Capability is classified as the psychological and physical abilities, such as knowledge and skills, that are needed to perform a behaviour. Opportunity includes the physical and social factors that enable or restrict the behaviour. Motivation, which includes the mental processes (reflective and automatic) that drive behaviour, is the central concept and mediates between behaviour on one hand and capability and opportunity on the other [19]. We used this framework to help contextualise our findings by juxtaposing individual competencies along with wider social and environmental opportunities that can influence participation behaviour. This framework was also chosen to methodically and clearly identify gaps and opportunities in migrants' participation in research, which could then inform appropriate and effective interventions to change that behaviour and improve participation.

## 3 Methods

We did a mixed‐methods study wherein we administered a paper‐based questionnaire in English to migrants and conducted focus groups and interviews with migrants and other key informants (KIs). We defined a migrant as anyone who was born outside the United Kingdom, a first‐generation migrant, irrespective of their current migrant status or length of stay in the United Kingdom.

### 3.1 Setting

The study was conducted in Leicester City, where more than 40% of the population was born outside the United Kingdom in 2023 [20]. Within Leicester, the study was conducted at two sites: Site 1 was an educational institution operating within the city that offers English for Speakers of Other Languages (ESOL) and other professional courses. We chose this setting because, from our previous research experience, we observed that the majority of ESOL learners are from a migrant background, which makes recruitment feasible. A charity organisation, that works mainly with asylum seekers and refugees, was included as an additional site (Site 2) for the qualitative study.

### 3.2 Sampling and Recruitment

For the questionnaire, we recruited migrant learners (≥ 16 years) from Site 1. To promote acceptability and uptake, questionnaire participants were recruited by gatekeepers at the institution who acted as the intermediaries between the research team and the participants. Questionnaire distribution occurred in an informal way, with students who expressed an interest being included. Therefore, we cannot provide a participation rate for this part of the study. We administered the questionnaire at Site 1 only after feedback from staff at Site 2 that their service users would have difficulty comprehending the questionnaire, and hence, the quality of data might be compromised. They suggested that face‐to‐face interactions with the researcher in a focus group setting would yield richer data. We also consulted gatekeepers at both sites about conducting the research in English, given our limited resources. While the gatekeepers agreed that language barriers could deter some migrants, particularly those who have recently arrived, they suggested that the wide variety of languages spoken by participants would make translation difficult. They also noted that since English commonly serves as the shared language among migrants, many already possess an adequate level of functional English, which we had also realised from our past research and engagement work [21].

Migrant participants for the qualitative study were recruited from both Sites 1 and 2. Staff and volunteers working at both sites were also included in the qualitative study as KIs. While KIs in Site 2 took part in a focus group, those in Site 1 were worried about inadvertently sharing confidential information about students in a focus group and therefore, opted for interviews.

### 3.3 Data Generation

The questionnaire items were developed through prompts from literature in the area and from our previous experiences in studies involving migrants. We pilot‐tested the questionnaire with a group of seven migrants at Site 1. After analysing the pilot results, we realised that the time taken for completing the questionnaire was significantly more (> 45 min), which is why we deleted some questions related to data sharing and instead added a question about digital literacy with a branching out question on data protection. We also changed the formatting of the questionnaire to make it user‐friendly.

For the qualitative study, two separate topic guides were developed for migrants and KIs, and broad topic areas covered in the questionnaire were explored, which included migrants' knowledge and perceptions about health research, prior experience of research participation, health needs and priorities, and motivations to take part. Additionally, the topic guide asked participants what they regarded as barriers to participation, and in the questionnaire, there were questions related to childcare, trust and preference for taking part in different types of research to understand how these factors can inhibit or facilitate participation.

Questionnaire data collection took place from October to December 2023, and questionnaires were distributed and completed by migrant learners in the classroom setting. Focus groups and KI interviews at both the recruitment sites took place from June 2023 to February 2024. All focus groups with migrants and KIs took place face‐to‐face, while interviews with KIs were either face‐to‐face or online, depending on participants' preferences. Focus groups and interviews were conducted by M.G. and recorded with prior permission from the participants.

### 3.4 Data Analysis

Qualitative analysis began with transcription of the interview and focus group recordings by a professional transcriber or generated on Microsoft Teams. Transcripts were first read by M.G. for familiarisation and recollection and from the initial reading it was realised that some of the data reflected what is available in literature about barriers to participation (e.g., language) while others are novel and nuanced in relation to our study participants (e.g., the opportunities available to take part in research). This made us decide to adopt the framework approach [22], which can be used to systematically but flexibly organise the data. M.G. did the first round of coding on a set of transcripts using NVivo software. Coding was both deductive, using some of the questions from the questionnaire as codes, and inductive from the data. The coding framework was shared with the team in weekly meetings. For the purpose of rigour, L.S. coded one focus group transcript using the coding framework, and any differences in coding were brought back to the discussion table. A working analytical framework was thereafter developed in discussion with the wider research team and applied by M.G. to the remaining transcripts. Any new codes that were identified from subsequent coding were included in the coding framework after discussion. In the next step, the data were charted into a matrix with summary categories. These categories were created by merging codes as a data reduction strategy. At this stage, we choose the COM‐B model as a framework for systematically organising the data categories. At the data interpretation stage, the team considered each of these categories individually and held discussions before arranging them under the three overarching themes of Capability, Opportunity and Motivation from the COM‐B model.

Quantitative data analysis was led by R.B.; data from the questionnaires were analysed descriptively using counts and percentages; and questions with Likert‐style responses (i.e., respondents rated their response using a scale) were summarised using Likert plots. Data were analysed using R (version 4.4.2). We integrated the quantitative data with the qualitative data at the data analysis stage by merging the qualitative and quantitative datasets and looking at these simultaneously to build a coherent narrative. At the presentation stage, integration was achieved by weaving in questionnaire items and outcomes to each of the three COM‐B themes from the qualitative analysis.

### 3.5 Public Involvement

Public involvement was critical to this study and this project. It started with inviting migrant members of the public to be part of a ‘Day in Research’ event in November 2023, wherein they attended a half‐day event at one of the hospitals in Leicester to give them an exposure to the range of research activities that are conducted in health research. Those interested in remaining involved were then invited to join as members of EMBRACE, the PPIE group. In February 2024, nine members of the group attended a workshop where preliminary findings of the study were presented using a role‐play that featured some of the initial themes. This was followed by discussions by the members, which were noted by the research team and reflected upon during the weekly team meetings. At the next meeting held in September 2024, we presented the COM‐B analysis along with the quantitative analysis and graphs to ensure that these are easily comprehensible. We also held discussions about the sub‐themes to decide which of the three components of the framework they would fit into, which helped with the final presentation of the qualitative data.

## 4 Results

Four focus groups with migrants were conducted (two at each site), with a total of 28 participants, while 119 migrants completed the questionnaire, all recruited from Site 1. Demographic characteristics of migrant participants stratified by study site and mode of participation are shown in Table 1. Further background characteristics collected from questionnaire participants are shown in Table S1.

**Table 1:** Demographic characteristics of migrants participating in focus groups for each of the study sites. For focus group participants (qualitative data), the number of participants (*n*) is shown; for questionnaire participants (quantitative data), both *n* and % are shown.

	Focus groups		Questionnaire	
	Site 1 (*n* = 12)	Site 2 (*n* = 16)^1^	Site 1 (*n* = 119)	
Median age, years (IQR)^1^	28 (19–32)	32 (27–38)	34 (27–40)	
Sex	Female (*n* = 12)	Female (*n* = 10), Male (*n* = 6)	Female (*n* = 97, 83%)^2^ Male (*n* = 20, 17%)	
Country of birth	Eritrea, India, Iraq, Latvia, Moldova, Somalia, Sudan or Uganda	Afghanistan, Cameroon, Ethiopia, India, Iran, Malawi, Sudan or Thailand	India Sudan Iraq Eritrea Somalia Bangladesh France Afghanistan Morocco Sri Lanka	(*n* = 18, 15%)^3^ (*n* = 16, 13%) (*n* = 12, 10%) (*n* = 10, 8%) (*n* = 6, 5%) (*n* = 6, 5%) (*n* = 4, 3%) (*n* = 4, 3%) (*n* = 4, 3%) (*n* = 4, 3%)
Ethnic group	Black African, Asian, White or Other	Black African, Asian or Mixed	Black/Black British Asian/Asian British White Mixed/multiple ethnic groups Other Prefer not to say/missing	(*n* = 38, 32%) (*n* = 35, 29%) (*n* = 24, 20%) (*n* = 2, 2%) (*n* = 14, 12%) (*n* = 6, 5%)
Length of stay in the United Kingdom	Range 1–18 years	Range 1 month to 7 years	Median 5 years, IQR 3–10 years, range 3 months to 30 years	
Current immigration status	Asylum seeker (*n* = 2) Refugee (*n* = 1) Indefinite leave to remain (*n* = 2) UK national (*n* = 2) Don't know (*n* = 5)	Asylum seeker (*n* = 13) Refugee (*n* = 2) Spousal visa (*n* = 1)	Asylum seeker/refugee Indefinite leave to remain UK national Dependent visa EEA national Work/study visa Don't know Other/prefer not to say/missing	(*n* = 30, 25%) (*n* = 24, 20%) (*n* = 24, 20%) (*n* = 11, 9%) (*n* = 18, 15%) (*n* = 0, 0%) (*n* = 4, 3%) (*n* = 8, 7%)

*Note:* Iran, Latvia, Poland and Ukraine (*n* = 2, 2%); Albania, Algeria, Cameroon, Russia (specifically Chechnya/Chechen Republic), Democratic Republic of Congo, Egypt, Ethiopia, Guinea, Kenya, Libya, Mongolia, Mozambique, Namibia, Romania, Saudi Arabia, Slovakia, Syria and Uganda (*n* = 1, 1%). Data on country of birth were not reported for three (3%) respondents.

^1^ IQR, Interquartile range.

^2^ Excludes one (1%) participant who preferred not to say.

^3^ Additional countries: Moldova and Pakistan (*n* = 3, 3%).

**Figure 2:**
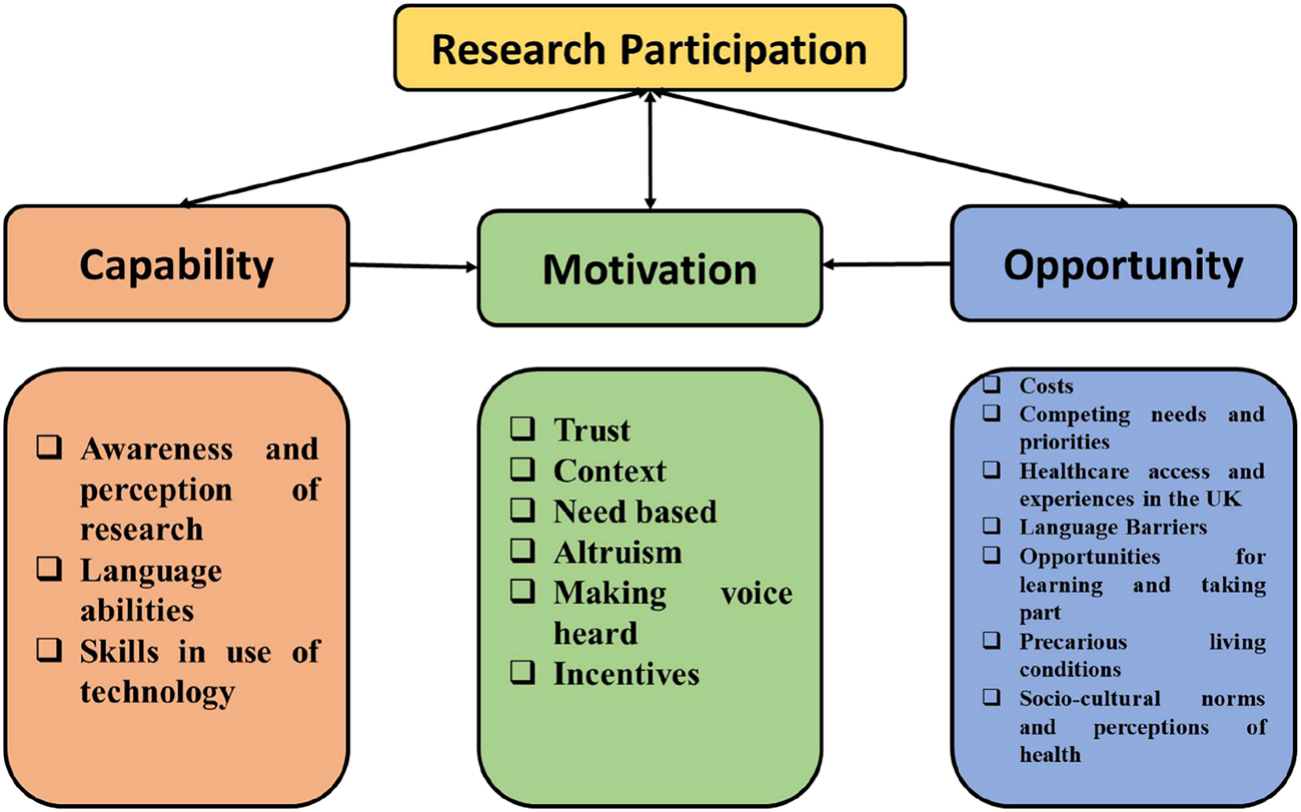
Diagrammatic representation of study themes and sub‐themes.

KIs at Site 1 opted to take part in individual interviews, and one focus group was conducted with KIs at Site 2. Seven KIs participated in individual interviews at Site 1 (all female), and seven participated in a focus group held at Site 2 (3 female, 4 male). Since both questionnaires and focus groups were conducted with migrant participants at Site 1, and there was no restriction on participation, a few participants participated in both. Three themes (and 16 sub‐themes) were developed from the qualitative data gathered from migrant participants and KIs (Figure 2), and descriptive statistics from the questionnaire data have been used to supplement the themes and sub‐themes. Illustrative quotes are provided for the sub‐themes in the text, and additional quotes are provided in Table S2.

### 4.1 Capability

Capabilities in the context of our study include the individual abilities, skills and/or knowledge that migrants have or don't have for taking part in research. These are:

#### 4.1.1 Awareness and Perception of Research

Awareness of research in general and health research in particular was found to be low among migrant participants. Nearly all migrant participants in the focus groups said they had not heard of health research and had never taken part in any research studies. KIs also said that very few migrants that they work with are aware of health research or have knowledge of it.

I: What do you think is a level of awareness or understanding about health research amongst your migrant learners?

KI, 1, Site 1: None. You have to really explain to them what happens, why the benefits of a research, of these studies. And that's how things can get better and you know doctors can look at ways to cure diseases and check where the gaps are. You have to really explain to them.

**Figure 3:**
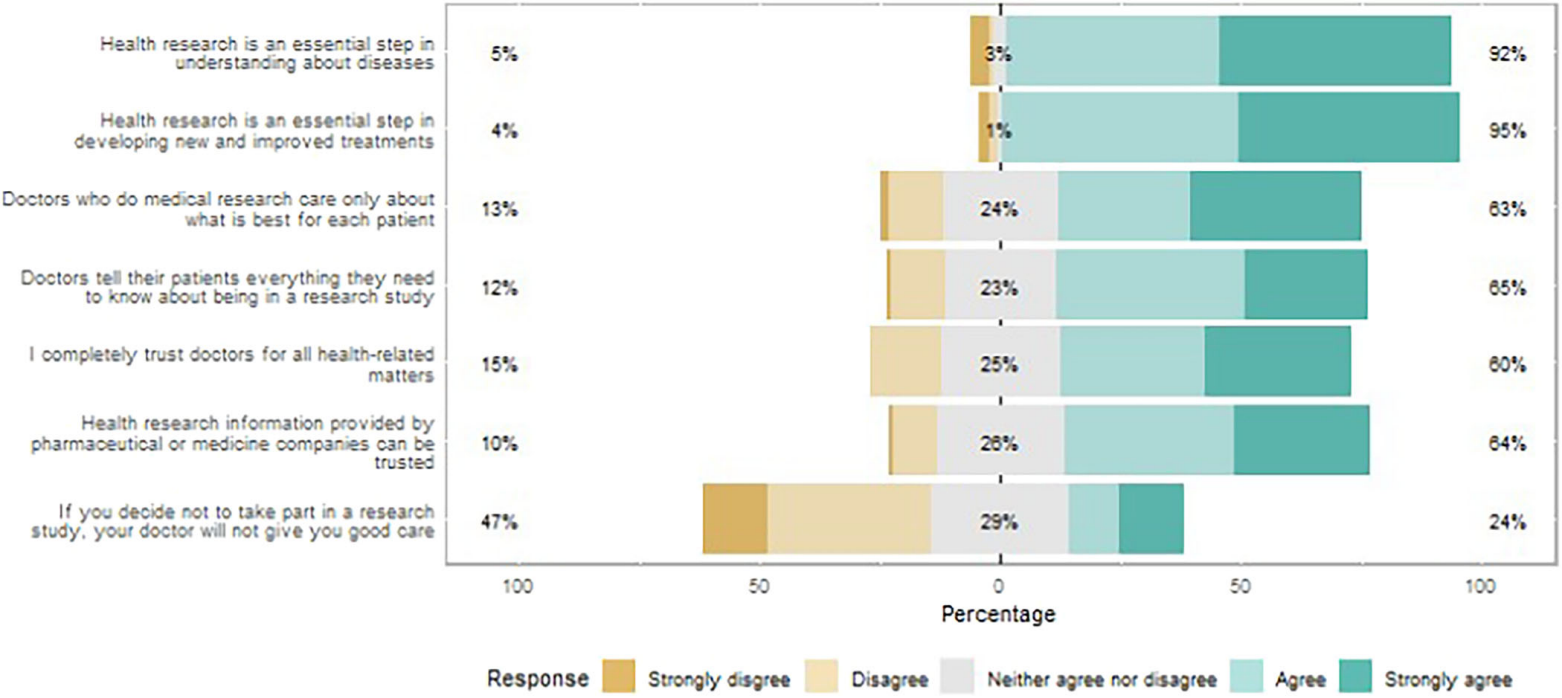
Questionnaire responses: trust in the healthcare practitioners, health research and health researchers. Percentages in the middle represent neutral answers (neither agree nor disagree); percentages on the right‐hand side represent positive answers (agree or strongly agree); percentages on the left‐hand side represent negative answers (disagree or strongly disagree).

There were similar findings among questionnaire respondents, and 76% of respondents said they had never taken part in research, and another 10% said they were not sure if they had. Similarly, nearly 85% (100/119) reported that they either did not know or were unsure about how a research study is done (Table 2). Interestingly, even though awareness about research was low, participants' perception of health research was positive and more than 90% of questionnaire respondents agreed that health research is an essential step ‘in understanding about diseases’ (92%) and ‘developing new and improved treatments’ (95%) (Figure 3).

**Table 2:** Questionnaire responses (*n* = 119) regarding views of and participation in health research, arranged by COM‐B framework themes (Capability, Opportunity and Motivation).

		*N* (%)
Capability		
Have you ever taken part in a research study?	Yes	15 (13%)
	No	90 (76%)
	Not sure	12 (10%)
	Missing	2 (2%)
If yes, can you remember what you had to do for the research study?	Talking about a topic	7 (47%)
	Taking medicine	0 (0%)
	Not sure/can't remember	6 (40%)
	Something else	1 (7%)
	Prefer not to say	1 (7%)
Do you feel you know much about how a health research study (e.g., testing a new vaccine) is run/what it does?	Yes	18 (15%)
	No	71 (60%)
	Not sure	29 (24%)
	Missing	1 (1%)
Would you be happy to take part in a research project using your smartphone/computer/laptop/tablet to complete questions (through the internet browser or through an app)?	Yes, definitely	55 (46%)
	Maybe/not sure	41 (34%)
	Definitely not	16 (13%)
	Missing	7 (6%)
Opportunity		
Do you have or care for children (< 18 years)?	Yes	66 (55%)
	No	49 (41%)
	Missing	4 (3%)
If yes: if you were to take part in a research study, do you have someone who could look after your children so you could attend the study without bringing them?	Yes	12 (18%)
	Perhaps, depending on the time of day of the study	8 (12%)
	Perhaps, depending on the duration away from home	5 (8%)
	Probably not	15 (23%)
	Definitely not	5 (8%)
	Not sure	18 (27%)
	Missing	3 (5%)
If you have a carer for your children while you took part in research, would you have to pay them?	Yes	16 (24%)
	Not sure	11 (17%)
	No	32 (48%)
	Prefer not to say	5 (8%)
	Missing	2 (3%)
Do you know that people taking part in health research are repaid any money they have to spend to take part in the research (e.g., travel costs and childcare costs)?	Yes	16 (13%)
	No	73 (61%)
	Not sure	27 (23%)
	Missing	3 (3%)
Would you be prepared to travel to any of the following places to take part in a research project?	Hospital	43 (36%)
	Local university	21 (18%)
	GP surgery	63 (53%)
	College currently attending	32 (27%)
	Local place of worship (e.g., church, temple and mosque)	10 (8%)
	Other community venues (e.g., community centre and library)	9 (8%)
Would you be happy to take part in a research project using your smartphone/computer/laptop/tablet to complete questions (through the internet browser or through an app)?	Yes, definitely	55 (46%)
	Maybe/not sure	41 (34%)
	Definitely not	16 (13%)
	Missing	7 (6%)
If not answering yes, reason why:	I don't have access to a gadget where I can use the internet or an app	10 (16%)
	I don't know how to use the internet or an app	9 (14%)
	I am worried about the safety of my data provided online	25 (39%)
	Other*	17 (27%)
	Missing	3 (5%)
Motivation		
I would be motivated to take part in health research because:		
	I want to help society	74 (62%)
	I want to learn more about diseases, illnesses and new treatments	55 (46%)
I would be motivated to take part in health research if:		
	A trusted member of my family/friends advises me to take part	49 (41%)
	A trusted member of my community (e.g., religious leader) advises me to take part	10 (8%)
	A trusted person at my college/workplace advises me to take part	32 (27%)
	My GP/doctor approaches me to take part	20 (17%)
	I know my participation and contribution will be valued and appreciated	34 (29%)

* All 17 respondents answering ‘Other’ wrote the reason as being ‘Hard Copy’.

#### 4.1.2 Language Abilities

Participants in the qualitative study suggested that English language skills and proficiency are important to enable understanding and participation in research. Conversely, some participants also said that not being able to speak English or not being proficient can make it challenging for them, not just in taking part in health research but also in their everyday lives. Some of our participants said that although they have basic English‐speaking skills, it is difficult when it comes to communicating about health issues, in particular, as that involves technical terms and specific terminologies.

I know how to read so if I have the form [Participant Information Sheet] I will read it first and if I think I will understand if safe [to take part] or not.

Female 1, Cameroon, 9 months in the United Kingdom

My friends, they have [health] appointment and they ask for interpreter and when they came for appointment, they don't have that interpreter and they struggle because sometimes you can speak English but you don't know all [terms] and it's hard for you to explain what you want.

Female 2, Latvia, 14 years in the United Kingdom

#### 4.1.3 Skills in Use of Technology

The use of technology, particularly the use of smartphones, has also been regarded by some qualitative participants as a skill which could promote migrants' participation in research. A few KIs observed how migrants, particularly those with limited English language skills, use their mobile phones and internet technology to access translated information, build and maintain networks and also navigate the various systems (health, education and immigration) that they interact with after coming to the United Kingdom. In the words of a KI:

So in my classes I do see [the use of technology] all the time. So sometimes if I come up with a new word or something, they immediately and I do allow them to use a mobile phone for obviously educational purposes … they all have translator apps on their phones. So they would use the translator apps to just find out the word and then understand and know how to pronounce the word. So yeah, they're pretty much good with using technology in that way.

KI 1, Site 1

However, some participants also alluded to the fact that a lack of these skills in the use of technology, along with a lack of access to technology, can disadvantage some migrants, which in turn can hinder research participation.

46% (55/119) of questionnaire respondents reported feeling confident about using technology in health research (reporting that they would definitely be happy to take part in a research project using their smartphone/computer/laptop/tablet to answer questions) (Table 2). 8% (10/119) reported that they could not participate digitally because they did not have access to a suitable device. A further 8% (9/119) reported that they did not know how to use the internet or an app, 21% (25/119) were worried about the safety of their data online and 14% (17/119) indicated that they would prefer to provide data on hard copies instead (which was the format of our questionnaire).

### 4.2 Opportunity

Opportunities, as described earlier, are the host of social and environmental factors that lie outside of an individual and can influence behaviour. The opportunities (or lack thereof) associated with migrants' participation in research, as identified in our data, include the following.

#### 4.2.1 Costs

Our data show that the costs incurred as a result of taking part in research can be a determining factor in research participation. These costs could be a loss of opportunity (opportunity costs) such as missing work, or missing children's school pick‐up/drop‐offs or financial costs (transaction costs) such as paying for travel.

…our [migrant learners] have to drop children before class, even for us … classes normally start at 9.15—even we have to allow a little bit of lateness because we know different schools … and then soon after the class they're rushing off to pick their kids up—so that's a massive barrier for them.

KI 4, Site 1

Speaking particularly about vulnerable migrants, another KI said:

I think the other thing is they [asylum seekers] do have a lot of appointments and they could be medical, but a lot of them are legal, things with their solicitors or they have to go somewhere to report … so there's a lot of things like that that make their lives quite erratic. If you're in a hotel you are given £8 a week, which is quite often a return ticket and you're only given like ten days, and if you miss it you really have consequences.

KI 5, Site 2

The majority of questionnaire respondents were carers for children (66/119, 55%, Table 2). Of these, only 12 (18%) answered confidently that they would have someone who could look after their children so they could attend the study without bringing them, with a further 13 (20%) possibly able to do so and 20 (30%) probably not or definitely not being able to find childcare and 16/66 (24%) stating that they would have to pay for childcare. Importantly, there was low knowledge that study participants are repaid any money spent in taking part in research, such as travel costs (only 16 [13%] reported that they were aware of this). KIs also highlighted the costs that community‐based organisations have to bear while facilitating research, which include staff costs, time away from their own assignments, venue costs and so forth.

#### 4.2.2 Competing Needs and Priorities

Several migrant participants said that they rank getting/working a job, safety, securing accommodation, and getting/completing education as higher priorities than health, in general. As a migrant participant shared:

I'm in the UK for a good life and to look after my family and I like to work.

Female 3, Iraq, 16 years in the United Kingdom

Another participant who declined an invitation to take part in a study when invited by his GP said:

Yeah, because I was busy with other things, I have different classes and the classes were very important for me or else I could take part.

Male 1, Origin not disclosed, 10 months in the United Kingdom

When these competing needs and priorities occur alongside costs and precarious living conditions, the motivation to take part in research could run quite low.

#### 4.2.3 Healthcare Access and Experiences in the United Kingdom

Most migrant participants spoke at length about their experiences of accessing healthcare in the United Kingdom and the challenges they face. These experiences, although not directly associated with research participation, can create or reduce trust in the system, which can influence participation in health research.

Speaking about long wait times and the frustration it creates, an asylum‐seeking participant with a life‐threatening diagnosis shared:

Before I also have to ring about my operation and somehow I want to kill myself in the last year and then they tell ‘OK, only emergency’ and after 3 months the GP has to ring me. They give me the pill to wait a long time.

Female 5, Thailand, 4.5 years in the United Kingdom

While negative experiences can alienate people, there are also opportunities for engagement with the health system and research for migrants who appreciate the care and treatment they receive in the United Kingdom. In the words of an asylum‐seeking participant:

When I first came here [UK] I think that I am satisfied for the way they treat me because when I first came I was traumatised, I had mental problems. They first bring me to the GP and the GP gave me mental care after they test blood, they have done all my exams. Everything, even about cancer, all over and I'm living in the hotel and they are paying for everything.

Female 1, Cameroon, 9 months in the United Kingdom

#### 4.2.4 Language Barriers

While we discussed language as an individual ability under the Capability theme, results reveal that the language in which the research is conducted could also be a (missed) opportunity for research participation. Of the 119 questionnaire respondents, only 20 (17%) reported English as the language most often spoken at home (See Table S1). Considering that most migrants come from non‐English‐speaking countries, participants said that having research information and procedures only in English could be a lost opportunity for them to take part. As one focus group participant said:

And then you are calling other people to take part in this research because there are lots of different cultures and different languages … and I'm sure lots of people have received maybe your email to take part in this research but it's been in English, not in their language, it would be very helpful.

Male 1, Origin not disclosed, 10 months in the United Kingdom

#### 4.2.5 Opportunities for Learning and Taking Part

The interviews and focus groups also highlighted the opportunities that are available to some of our migrant participants to learn about health issues, the healthcare system, and also health research in their everyday lives. Many of the KIs at Site 1 shared about discussing topics like accessing the NHS, diet and health, vaccination and so forth with their migrant learners in the classroom. They also mentioned how migrants depend on social networks and connections to acquire information about health systems and processes. KIs at Site 2 also talked about conducting sessions on health screening and vaccination, as part of awareness generation amongst newly arrived migrants.

We cover the topic of health throughout our teaching. I mean mainly it is good for the learners, this is how this skills for life was devised, right? … For example, at a lower level, it would be just being able to make an appointment, calling the doctor, calling for an emergency service and asking for a medication…. At the higher level, maybe applying for jobs in healthcare if they're interested or volunteering in healthcare or, being aware of what to do, look at the NHS websites and depending on what progression they want to do.

KI 7, Site 1

These opportunities for learning for migrants also mean that there are opportunities for researchers to promote research and facilitate migrants' access to research.

Physical spaces are also important for research access, and the questionnaire asked respondents if they would be prepared to travel to different venues to take part in a research project. GP was the most common choice (56%), followed by hospital (36%) and their current place of learning (27%). Interesting community venues such as places of worship (8%) and community centres (8%) were not highly preferred.

#### 4.2.6 Precarious Living Conditions

Many participants in our study spoke of the precarious living conditions that come with being vulnerable migrants, like asylum seekers and refugees, which is likely to minimise the opportunities they have to take part in research. Several participants shared that they faced an existential crisis after arriving in the United Kingdom, not knowing if they would be allowed to stay, where they would live, what living conditions they would have, and so forth. KIs have also highlighted that ironically, the precarity increases when people are granted refugee status, as then the support received from the Home Office is no longer available. As one refugee participant said:

Yes, the waiting [for a decision] is like a big torture for everyone, like brain torture, like soul torture … you are stuck in one room for years, years, years, years, and you can't move from that. If you want to go to see your friends or family for other cities, you don't have the money to go there and to visit them…. Why escape from my country to come here to have my own freedoms?

Male 3, Afghanistan, 7 years in the United Kingdom

#### 4.2.7 Socio‐Cultural Norms and Health‐Seeking Behaviour

Migrants come from varied cultural backgrounds, and participants hinted that sometimes the socio‐cultural norms and obligations can inhibit free choices, including the choice to take part in research, and this was specifically mentioned in the context of women. As a KI stated:

Some of them *[migrant female learners],* in fact majority of them, will not say yes to anything unless it's approved by their partners at home … even if it's not partners – other family members like parents‐in‐law—they also have a say in a lot that goes on in their daughters‐in‐law. Yeah, so that for me is just another really big barrier….

KI 9, Site 1

Speaking about cultural restrictions, another KI said:

Also, some cultures will not take part in medical research because it's classed as something that's not appropriate. Communities that I've worked with before prefer to either see their own healers or somebody within their own community. They won't take part in anything that's outside of their community.

KI 4, Site 1

Participants also shared how culture shapes people's perceptions about health and the importance of health. Speaking of her own community, a migrant participant shared about delayed healthcare seeking:

Yeah, also some people, they have problem, issue, problem, but they don't like to go to hospital, but after the health like increase, they go to their GP or hospital and the issues like become increased….

Female 4, Eretria, 4 years in the United Kingdom

### 4.3 Motivation

Motivation can be deliberate or automatic, and in the context of research participation behaviour among migrants, the following motivations have been found to help or deter participants from engaging in that behaviour.

#### 4.3.1 Trust

Trust operated in two separate but interlinked contexts: trust in the research and trust in who is approaching them to take part in the research. Trust in research was mainly in relation to issues about safety, security and data protection. Trust was also associated with people's past experiences in their home countries and also suspicion that research data may be used for surveillance. As one asylum‐seeking participant said:

It *[research participation]* depends, if it's not going to bring me trouble…. Trouble like, you know, my background we don't have sometimes the opportunity to give our opinions in my country so we just do what we have to do. This is a new country, I don't know how it works here so I have the same fear that I was having in my country, so I don't allow to say something, to go. So I don't know, that's what I'm afraid of. Here I don't have, you know my situation so I don't want to ruin it.

Female 1, Cameroon, 9 months in the United Kingdom

In terms of trust in who is approaching them to take part in research, KIs shared that they have observed from their interactions with migrants that over time the trust of their users on them increases and they will take their word.

Trust is the critical thing. If you have an asylum seeker coming in and I come and say ‘hey, talk to these guys’, they don't know you, they're almost certainly going to be on the back foot, defensive because of what they had been through. It's only after a bit of time with us that people have the opportunity to open up and you get to the point where people will trust the people who say it's OK to trust because it's you saying it, if that makes any sense.

KI 10, Site 2

Questionnaire respondents reported relatively high levels of trust in healthcare practitioners and health researchers (Figure 3), but around a quarter of respondents neither agreed nor disagreed with statements regarding the integrity of doctors and pharmaceutical companies, and a substantial proportion disagreed (10%–15%). Nearly a quarter (24%) of respondents agreed or strongly agreed with the statement that, ‘if you decide not to take part in a research study, your doctor will not give you good care’, demonstrating the need for reassurance regarding this to be included in all invitations to take part in health research. Interestingly, 41% of respondents reported that they would be motivated to participate in health research if a trusted family member/friend advised them to take part, and 27% said that they would be motivated if a member of their college or workplace advised them. 17% respondents stated that they would be motivated to take part in health research if their GP/doctor approached them to take part (Table 2).

#### 4.3.2 Context

The context of the research was also found to motivate or deter people to join. While participants said that they would be willing to take part in questionnaire surveys or qualitative studies, the same enthusiasm was not there for certain other types of research, such as clinical trials of vaccines or medicines. Risks associated with research which involves ingesting or injecting something were mentioned as reasons for not wanting to take part in such studies. When asked about whether they would take part in a new vaccine trial as an example, a participant said:

It would be risky…. You may get death or something…. You always have to sign and agree and if anything happens oh it will be your responsibility.

Female 6, Somalia, 18 years in the United Kingdom

Similarly, a KI remarked:

I think if it's to draw blood, you know, I don't think they [migrant learners] would mind so much. But having something in their body…. That's a different thing.

KI 1, Site 1

**Figure 4:**
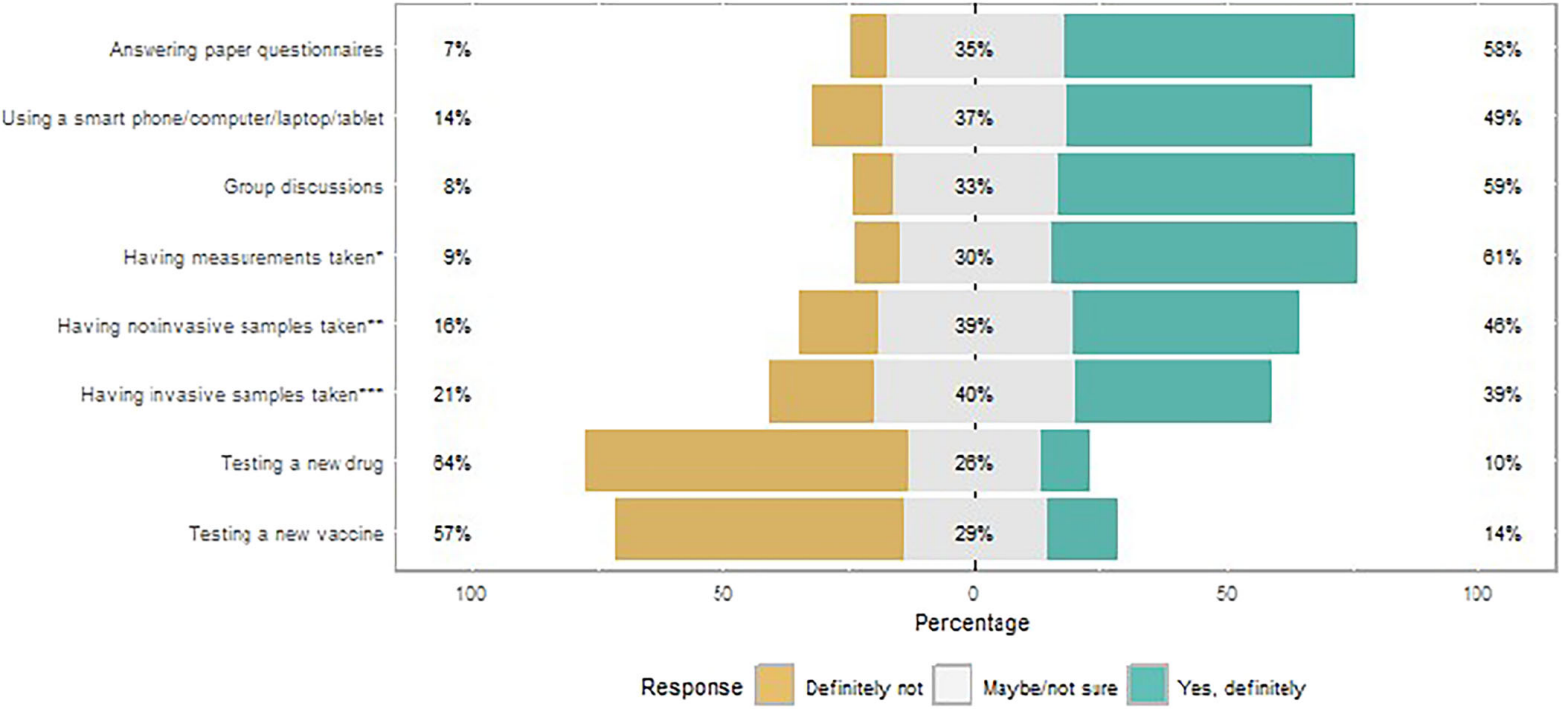
Questionnaire responses: willingness to take part in health research studies according to activities involved. Percentages in the middle represent neutral answers (neither agree not disagree); percentages on the right‐hand side represent positive answers (agree or strongly agree); percentages on the left‐hand side represent negative answers (disagree of strongly disagree). *Weight, height, **Urine sample, ***Blood sample.

The same patterns were observed in questionnaire responses, with fewer respondents willing to participate in research studies which became more invasive (16% and 21% unwilling to have non‐invasive and invasive samples taken, respectively, compared to 7% unwilling to answer paper questionnaires, Figure 4). A much larger proportion were unwilling to participate in drug and vaccine trials (64% and 57%, respectively).

#### 4.3.3 Need Based

If the research meets the needs of the participants, the likelihood of them taking part increases and vice versa. In the words of a migrant participant:

…if the people have a need, they will do the things you want to get from them … otherwise it's a little bit difficult—everyone has their problems—their things to do and they don't want to spend time going somewhere, because they are not interested about this research.

Female 8, Moldova, 5 years in the United Kingdom

This was also echoed by a KI who said:

So now imagine asking them to go for research because obviously it's never occurred to them that it's a need. So, it has to shout out need—you need this otherwise you won't be able to do this. If it's something that doesn't sound like a need it's going to be difficult.

KI 3, Site 1

#### 4.3.4 Altruism

Altruism or the understanding that their participation could potentially lead to well‐being of others was also regarded as a motivating factor by some participants. When discussing the reasons behind their taking part in health research in the focus groups, participants said:

To find which way is better to help everyone and find everything is good.

Female 3, Iraq, 16 years in the United Kingdom

For the community, to help improve the health.

Female 6, Somalia, 18 years in the United Kingdom

Similarly, 74 (62%) of questionnaire respondents stated that they would be motivated to take part in health research because they want to help society, and 34 (29%) stated that they would take part if they knew that their participation and contribution would be valued and appreciated (Table 2).

#### 4.3.5 Making Voice Heard

While altruism was a consideration for some participants to take part, some others said that they would be motivated because of the opportunity research can provide to make their voices heard. As one participant said:

If people do that research questionnaire, I think they do that because they have a lot to say and they want to say that and to be heard.

Female 8, Moldova, 5 years in the United Kingdom

Speaking of how the desire to make oneself heard can motivate migrants to take part in research, a KI shared:

*[taking part in research]* would be very beneficial for our students and also empowering as well because a lot of students say that they feel that they're not important, they're not listened to, people don't understand.

KI 4, Site 1

#### 4.3.6 Incentives

Focus group participants shared various incentives (monetary and non‐monetary) that could act as motivations for them to take part in the research. While a few participants said that getting vouchers or payments is helpful, many others said that they would consider participating because it would help them get new and additional information about diseases and conditions (including nearly half, 55/119 [46%] of questionnaire respondents, Table 2), or offer them opportunities to practice their English‐speaking skills, and generally as helping them in their future.

Because it asks about our health I can know and doctor can tell us what we have to do if we have some diseases or something.

Female 7, Eritrea, 3 years in the United Kingdom

If you want to do another research this [shopping voucher] is a motivation for people as well because these people are asylum seekers, they need something to motivate because if you want, of course everyone wants to help someone. If they provide something, the people motivate more people.

Male 2, Afghanistan, 7 months in the United Kingdom

## 5 Discussion

Our study is one of the few pieces of research conducted with migrants in a high‐income country to understand their perceptions about participation in health research. We used the COM‐B framework to explicate the range of factors and conditions that can influence research participation behaviour among migrants. We have grounded our analysis in the COM‐B framework, against following the binary and linear logic offered by the ‘barriers and facilitators’ approach, to elucidate the complex systems and processes that can influence research participation behaviour among migrants [23]. Our data show how individual capabilities (C), combined with opportunities (or lack therefore) (O) created by environmental and social factors can influence migrants' motivations (M) or decisions to engage in research participation behaviour.

Awareness and knowledge of health research were found to be low among participants in our study. This could likely be due to the fact that exposure to health research has been limited for most of our participants who have come from low‐ and middle‐income countries where health research is still underdeveloped, mainly due to political and economic reasons [24]. The experiences of our participants with the healthcare systems in their home countries have mostly been in the context of a service provider, and this potentially continues to influence their relationship with the health system in the United Kingdom. However, it is also interesting to note that although we had participants in our study who have lived in the United Kingdom for several years now, knowledge and awareness about health research were still low. This indicates that communication about health research is not reaching many groups, and innovative communication channels such as community radio, places of worship and so forth need to be utilised more for greater outreach and higher engagement [25]. This also closely relates to our finding that there are insufficient opportunities for ethnic and migrant groups to take part in research. As Wendler et al. report from their analysis of 20 interventional and non‐interventional health studies, conducted mostly in the United States, that ethnic minority participants are not less willing to take part in research, but rather less likely to be invited to take part [26]. It is, therefore, increasingly argued that these groups are not ‘hard‐to‐reach’ [27] but rather it is our research and recruitment processes which pose barriers to recruiting these underserved groups in research. This has led to a proliferation of research to test and implement different strategies for recruiting migrant participants into health studies [28, 29, 30, 31, 32, 33]. Researchers working with underserved groups need to consider these strategies while designing their studies.

Language as a barrier to research participation has been explored in previous studies, mainly in the context of racial or ethnic minorities [34, 35, 36]. Different settings/countries will have different dominant languages (e.g., German in Germany), and depending on which language an individual migrant speaks they may either find this as a barrier or facilitator [37]. For example, a migrant who comes from a French‐speaking nation in Africa is likely to face fewer linguistic challenges in France than they do in the United Kingdom, where the dominant language is English. However, it would be wrong to assume that language barriers in research are purely participant‐related factors. We have highlighted through our analysis how the lack of multilingual information in research for non‐English‐speaking migrants is actually a missed opportunity created by factors (e.g., faults in research design, costs of translation, etc.) outside the control of an individual participant. Health research is usually text‐laden, with consent forms and participant information sheets often relying on people's ability to read and comprehend these documents. Hence, not having research information in non‐English languages, or even poorly translated material, can restrict participation, create confusion, fear and mistrust, and also be regarded as a sign of disrespect by migrant participants [34, 36]. This is an important consideration for researchers working with multicultural and multi‐ethnic groups.

Technology is pervasive in modern life, and a growing number of health studies are using technology for data collection, such as online surveys. While the use of technology can greatly benefit the researcher, concerns have also been raised about the biases it creates owing to the ‘narrowing but deepening’ digital divide even in high‐income countries such as the United States and the United Kingdom [38, 39, 40]. Our data reiterate the digital divide by stating how certain groups of migrants may be excluded from digitally conducted research either because they do not have the skills or cannot afford digital services or do not trust the safety of online platforms. This finding also highlights the need for further research on understanding digital health literacy needs and competencies of these underserved populations and how these can be improved/optimised.

The costs that come with taking part in health research might not always be visible and can be in the form of lost opportunities, as highlighted in our study. While costs as a barrier to research participation might be applicable to the general population as well, these may be more pronounced and exaggerated for migrants because of their need to work (as many countries, including the United Kingdom, do not allow recourse to public funds to economic migrants) and/or lack of social support (which may impinge upon child care or other care duties). Offering financial incentives for participation could balance out some of these costs, but payments in research have been a controversial topic and also fraught with problems [41, 42]. One of the arguments against making monetary payments to participants is that it could coerce participants to take part [43]. Our research, however, clearly shows that participants might not always be motivated by monetary payments, particularly when incentives are nominal, and knowledge enhancement can also be regarded as a benefit of research participation. This motivation may arise out of the social, economic and career aspirations that migrants have and indicates that they may be attracted to research participation by non‐monetary gains as well. Similarly, the desire to share their voices and help others by their participation have also been found to motivate our participants. However, as described in literature, altruistic motivations may not always lead to research participation, unless people see the research as fulfilling an individual need or benefiting them personally [44]. This links closely to our other finding about need‐based motivation, where participants shared that if the research fulfils their need, the motivation to take part is likely to be stronger. Conversely, as most newly arrived migrants are healthy and have limited health needs [3], the motivation to take part in health research for personal benefits could therefore be low.

Another significant impediment to research participation that was identified in our study was the precarity of living conditions that some migrants face. Although this finding was specific to marginalised migrants in our study, it could also be true for other underserved groups, such as homeless people [45]. Previous research has highlighted problems with recruiting migrants living in precarious conditions and recommended strategies such as building community relationships, using snowball sampling, cultural adaptation of recruitment material, and building the cultural sensitivity and competency of the research team [8, 46].

Our results also indicate that gender norms and religious and cultural beliefs that are part of many non‐Western societies can influence research participation among migrants. Strict patriarchal practices in certain cultures can reduce women's agency to decide to take part in research and lower their chances of participation [47, 48, 49]. Religious beliefs can also influence participation, especially in clinical trials that involve medical products derived from animals that are prohibited or procedures which are not sanctioned by their religion [50]. Researchers working with migrants need to be aware of these aspects so these can be factored into the planning and recruitment strategies.

Our findings also clearly demonstrate that participation in certain kinds of research such as vaccine development studies may be impacted by considerations of risk. While the potential risks involved in certain clinical trials cannot be negated, clear and consistent communication about risks and benefits, support with answering questions and patient's own state of health can support with decision‐making and building trust [51, 52, 53].

Trust is a key determining factor in research involving human participants. McDonald et al. [54] explored how trust works in motivating people to take part in research and found that trust is multilayered, involving both abstract and concrete choices (e.g., trusting research integrity of educational institutions and not trusting pharmaceutical companies), which are rooted in general perceptions rather than specific experiences. Trust is also dynamic and can be fostered or broken through an interpersonal relationship between the researcher and participants and involves an element of negotiation [54]. Guillemin et al. reaffirm that participants have trust in certain research institutions, such as universities, due to the strict research governance processes followed at these institutions [55]. Evidence also suggests that family and friends are often the most trusted sources of health information for many people [56], which explains our finding that many participants would be motivated to take part in research if they are advised by their friends or family. Furthermore, in their research with indigenous communities in Australia, Guillemin et al. also found that decisions to participate in research by these communities were determined by demonstration of honesty, reciprocity and respect by the research team [57]. These lessons are equally important in the context of working with migrants, where efforts to respectfully and meaningfully engage participants by researchers can go a long way in securing trust and encouraging participation.

Our study highlights a range of factors that could influence the research participation behaviour of migrants. These findings are comprehensive and reiterate the argument that people are not ‘hard‐to‐reach’, but it is the processes that create barriers to participation. Our research recommends creating awareness about research, optimising opportunities for promoting research in non‐clinical settings, improving digital health literacy, building relationships of trust, and working with trusted gatekeepers to improve the participation of migrants in health research. These recommendations are also applicable while working with other underserved populations, such as ethnic minorities.

### 5.1 Strengths and Limitations

One of the limitations of our study was that the research was conducted in English, and the inclusion criteria for taking part were to have functional‐level English speaking skills. While we do acknowledge that this could have potentially led to bias in the selection of participants and limited generalisability, for some research methods, like focus groups, it's especially challenging to include multiple languages as it makes social interaction, which is the essence of focus groups, quite difficult. Demographic data of our participants reflect the diversity of countries and languages spoken by our participants, and it would have been logistically difficult to organise and conduct the focus groups with interpreters in all the languages. Furthermore, as the research was conducted in a single UK city, which is ethnically diverse with a substantial migrant population, experiences of migrants in other parts of the country, particularly where immigrant numbers are low, could be different. The findings of this study should therefore not be used to draw general conclusions but instead can lay the foundation for larger studies with representative samples.

Another potential limitation of our study is that, because the questionnaire was administered in a classroom setting, respondents may have discussed their answers with each other. We chose this method to help participants understand the questions if needed and to reduce non‐response. However, based on the response patterns, we believe most respondents answered independently, with minimal group influence.

A key strength of our project was our robust engagement strategy. We established trust between the research team and the community by engaging gatekeepers at both sites early on, building rapport through non‐research activities, securing endorsements from trusted individuals, and involving researchers from diverse ethnic and minority backgrounds that participants could identify with [58]. Public involvement mainly occurred during data analysis and interpretation. Following established approaches [59] (Stocker et al. 2021), we used role‐play and interactive activities, along with written theme summaries, to share our results and foster organic, meaningful engagement with the group.

## 6 Conclusion

Our study contributes to the limited evidence base exploring migrants' participation in health research. Our findings, grounded in the COM‐B model, exhibit how migrants' motivations, influenced by a host of individual capabilities and environmental and social opportunities, can impact research participation behaviour. These findings are highly relevant for health researchers, who should consider the interaction of these factors across the three domains (COM) while designing their research to make it more accessible, inclusive, equitable and impactful. Although our findings are presented in the context of migrants, most of the factors are also applicable to other underserved population groups such as ethnic minorities.

## Author Contributions

**Mayuri Gogoi:** conceptualisation, writing – original draft, methodology, writing – review and editing, project administration, data curation, formal analysis, visualisation, investigation, funding acquisition. **Rebecca F. Baggaley:** conceptualisation, methodology, formal analysis, writing – original draft, writing – review and editing, visualisation, data curation, investigation, funding acquisition. **Luisa Silva:** writing – review and editing, formal analysis. **Zainab Lal:** writing – review and editing, formal analysis, investigation. **Holly Reilly:** project administration, writing – review and editing, investigation. **Vishant Modhwadia:** project administration, writing – review and editing, data curation. **Daniel Pan:** writing – review and editing. **Paul Bird:** writing – review and editing. **Laura Nellums:** conceptualisation, methodology, writing – review and editing, supervision, funding acquisition. **Manish Pareek:** conceptualisation, funding acquisition, writing – review and editing, supervision.

## Disclosure

The views expressed in this publication are those of the authors and not necessarily those of the NIHR or the Department of Health and Social Care.

## Ethics Statement

Ethical approval for the study was given by the Medicine and Biological Sciences Research Ethics Committee, University of Leicester (Reference Number: 38240‐mg432‐ls:respiratorysciences).

## Conflicts of Interest

M.P. reports grants from Gilead, Sanofi and Moderna and has received consulting fees from QIAGEN and Gilead. The other authors declare no conflicts of interest.

## Supporting information

IMMERSE Supporting Material 11.

## Data Availability

The data that support the findings of this study are available upon request from the corresponding author. The data are not publicly available due to privacy or ethical restrictions.
