# Supplementary material for: Migrants Living in the United Kingdom and Their Perceptions of Participation in Health Research: A Mixed‐Methods Study
Source: Health Expect. 2025 Jun 30;28(4):e70337. doi: 10.1111/hex.70337 (PMC12207088; doi:10.1111/hex.70337)
Supplement: Supplementary file 1 — IMMERSE Supporting Material 11. [file HEX-28-e70337-s001.docx]

**Supplementary Table 1:** Additional background characteristics for questionnaire participants

|  | | **N (%)** |
| --- | --- | --- |
| Religion | Muslim | 77 (65%) |
|  | Christian | 21 (18%) |
|  | Hindu | 12 (10%) |
|  | Sikh | 2 (2%) |
|  | Jewish | 0 (0%) |
|  | Buddhist | 0 (0%) |
|  | Other/Prefer not to say/Missing | 6 (5%) |
| Highest education level^1^ | No formal education | 3 (3%) |
|  | Primary school | 5 (4%) |
|  | Secondary school up to 16 years (e.g., GCSEs) | 18 (15%) |
|  | Higher secondary/further education (e.g., A Levels) | 28 (24%) |
|  | College or university undergraduate degree | 31 (26%) |
|  | Postgraduate degree | 6 (5%) |
|  | Other/Prefer not to say/Missing | 28 (24%) |
| Lived in country other than UK and country of birth | Yes | 28 (24%) |
|  | No | 87 (73%) |
|  | Prefer not to say/Missing | 4 (3%) |
| Employment status | At school | 24 (20%) |
|  | At university | 24 (20%) |
|  | Self-employed | 0 (0%) |
|  | Part-time employment | 18 (15%) |
|  | Full-time employment | 30 (25%) |
|  | Unable to work due to disability | 0 (0%) |
|  | Homemaker/full-time parent | 11 (9%) |
|  | Not eligible to work^2^ | 4 (3%) |
|  | Unemployed and seeking work | 3 (3%) |
|  | Retired | 4 (3%) |
|  | Prefer not to say | 0 (0%) |
| Language spoken at home most of the time^3^ | Arabic | 29 (24%) |
|  | English | 20 (17%) |
|  | Gujarati | 16 (13%) |
|  | Kurdish | 16 (13%) |
|  | Somali | 7 (6%) |
|  | Bengali | 6 (5%) |
|  | Urdu | 6 (5%) |
|  | French | 6 (5%) |
|  | Hindi | 5 (4%) |
|  | Romanian | 4 (3%) |
|  | Tamil | 4 (3%) |
|  | Tigrigna | 4 (3%) |
|  | Other^4^ | 46 (39%) |
| Registered with a GP | Yes | 112 (94%) |
|  | No | 3 (3%) |
|  | Don’t know/Missing | 4 (3%) |
| Long-term health condition | Yes | 20 (17%) |
|  | No | 94 (79%) |
|  | Prefer not to say/Missing | 5 (4%) |

^1^ Excluding attendance at Site 1.

^2^ Not eligible to work due to immigration status e.g., asylum seeker.

^3^ Many respondents (17%, 20/119) reported more than one language and so numbers total more than 119. Of these, 12 (60%) reported speaking English with one other language.

^4^ Additional languages: Polish (n=3, 3%); Amharic and Russian (n=2, 2%); Albanian, Dari, Italian, Lingalo, Mongolian, Otjiherero, Parsi, Pasklo, Portugese, Punjabi, Rufumbira, Tegreya and Ukrainian (n=1, 1%); and Chinese (n=0, 0%).

**Supplementary Table 2: Illustrative quotes under different sub-themes**

| **Theme** | **Sub-themes** | **Illustrative quotes** |
| --- | --- | --- |
| Capability | Awareness and perception of research | *My guess would be like very little* [understanding of research] *and potentially they might think health research is about being prodded and poked and being given medicines.* [KI 2, Site 1] |
|  | Language abilities | *…some of the more established* [migrants]*, I think those people have all had experience of the health system for better or worse. I mean they'd be very keen I think to be involved in research because they understand what it’s about, they understand what’s worked for them, what hasn’t worked for them so well and I think they're very skilled, because they’ve got good English, that’s the other issue, I think.* [KI 3, Site 2] |
|  | Skills in use of technology | *Usually before I go to my appointment where I need to discuss what I am concerned, if I don’t know some words in English, before I go I use Google Translator, write in my language, translate to English, write on the paper and when I go to appointment I start to talk with doctor and I say to him what I was translated.* [Female 2, Latvia, 14 years in UK]  *Low level IT skills are also an issue. And no access to IT equipment. So now everything's online. Registering with your GP. Registering for benefits. Now people are in a position where they either have to pay somebody who delivers that service within the local area or they have, or they have to ask a friend for help. So that is a big issue.* [KI 4, Site 1] |
|  | | |
| Opportunity | Costs | *So that in itself the distance and then the cost of buses and the restrictions of like you say, so childcare or even just the school pickups and drop offs does make things tricky for them.* [KI 2, Site 1]  *Well, what we notice is that students stop attending college and because they've got so many appointments that they've got to go to, they no longer can continue with their studies.* [KI 4, Site 1]  *I think we often have grants that money comes to enable x, y, z activity, but things like staff costs and food and all the basic things that makes it work isn’t included. It also has an impact on the way that we work and the pressures on us.* [KI 3, Site 2] |
|  | Competing needs and priorities | *Getting to work and whatnot. Yeah, but at the moment, even I have to deal with these kinds of situations where I have students, ‘Sorry I can't stay for the whole session. I need to leave at this time because I have to get to work’. And there is nothing that we can do about it, yeah.* [KI 1, Site 1]  *It* [taking part in research] *depends on the day because some of us like are working.* [Female 4, Eretria, 4years in the UK]  *If I have time of course I will* [take part] *because I know this research, it’s about something to improve the system of NHS or improve the system of health, so of course I will do that*. [Male 2, Afghanistan, 7months in the UK]  *I think the challenges are it's the practical things of how of how you do it and. For us as teachers, if we facilitate it, we've got quite time pressured courses and so losing even an hour of a class if you're in a short term, you'd be quite reluctant to do that because it means dropping something else.* [KI2, Site 1] |
|  | Healthcare access and experiences in the UK | *They've* [forced migrants] *already arrived with severe anxiety or post-traumatic stress disorder, and then it's exacerbated because they're struggling to engage with services and the processes that we have in the UK make it very difficult for them. And that's where people start to disengage.* [KI 4, Site 1] |
|  | Language Barriers | *It’s* [language] *absolutely vital. Because how can they even understand the questions, unless you have interpreters of course, if you have interpreters then language is not so important, but you still have to have something in all their community languages to attract them in so that they understand enough to take part in the first place..*. [KI 6, Site 1] |
|  | Opportunities for learning and taking part | *Yeah, we would encourage them to take part in anything…we do our own course rep surveys online…regarding health wise, teachers teach topics around health. Our teacher reading class on a Monday morning and this term I've been doing parts of the body and what do we need to keep fit and healthy.* [KI 8, Site 1] |
|  | Precarious living conditions | *Another thing to mention is a lot of people, especially if they* [asylum seekers] *haven't been here very long, might live life one week at a time, because you don’t know, am I going to get an interview in a week’s time, am I going to get sent here in a week’s time.* [KI 5, Site 2]  *And then also those, maybe those who are asylum seekers, so they're kind of living in limbo, they're waiting to find out what's gonna happen. And that means as well that they're they can be moved like within a week. And we had that last term. We had a number of students that one week they were with us and the next week they'd moved to a different city. So there's quite a lot of instability for asylum seekers in that way. Yes, I think those are there's a kind of a lot going on. And then of course, whatever their, the state of their family relationships back home. So for some, obviously, they're living in difficult circumstances. If they're in Ukraine. And then for others they're providing for family back home. So yeah, that impacts their priorities.* [KI 2, Site 1]  *…the health needs of the students are we have asylum seekers, we have students that once they move from asylum to refugee status, they become homeless, which is a difficulty for them. We had a student who was homeless who was taking things out of bins and he became very sick in class so I had to take him and his friend to the health centre…We had a student that actually did commit suicide because of his mental health and what was going on…Yeah, the health needs of the students are some of them are living in dire conditions even though they’ve got housing which affects their health a lot, yeah.* [KI 8, Site 1] |
|  | Socio-cultural norms and perceptions of health | *For example, it might be a woman wanting to take part, but then if the husband says no, just an example, I'm not biased when I'm saying women, you know, I know, allowed or what. But just an example. Then of course they might not,* [KI 7, Site 1]  *I think it depends on their own experience of healthcare, they might not think they might not realise like what like why should I bother if I'm healthy? Like what does it, what does it matter? And so understanding that actually it's useful to get the data from healthy people as well.* [KI 2, Site 1]  *…when I die, I don’t need the eyes, kidney, heart, but it’s my religion don’t allow [organ donation] because…when you are my eyes for you, you’re looking anywhere bad, my eyes are now in you and are your eyes…My opinion is it’s OK, but when my religion says no…I can donate blood, blood for everyone…It’s not a problem because every time blood is changing. Not forever. Every month it’s changed blood…It’s not a problem for donating blood.* [Female 3, Iraq, 16years in the UK] |
|  | | |
| Motivation | Trust | *Yeah, they* [migrant learners] *do ask. But how they're gonna use these? Are they gonna share? With whom they're gonna share? Can we put a fake address? Can we put a fake number…they do talk about what if it's hacked and what if this and there is always the ‘what if’. I don't know whether it's because they hear things, you know in the news or maybe social media, you know, of hacking, of personal data.* [KI 1, Site 1] |
|  | Context | *For example, it’s maybe cream* [ointment] *or you can put like somewhere small part of your body, I will be agree, but for drink or for like go inside* (shaking head to say no) [Female 7, Eritrea, 3 years in UK] |
|  | Need-based | …*some people who have serious illness, for example cancer and no-one can help them and they will try some new pills or some new treatment or I don’t know what, and they say yes, they want to try that because anyway, he tried to keep alive because there are no other options.* [Female 2, Latvia, 14 years in UK] |
|  | Altruism | *We like to help everyone by learning something new.* [Female 9, Uganda, 1 year in the UK]  *I think they would take part in the research if they believed they can help other people. I think that is a human thing and that we have deeply spiritual – some of these people are deeply spiritual and that matters to them I think so and that is the ultimate motivation.* [KI 6, Site 1] |
|  | Making voice heard | *To get heard. To get more information about how the health system is working or how to improve the health in the UK.* [Female 6, Somalia, 18years in the UK] |
|  | Incentives | *I think that if they're getting a voucher as well they'd probably be very pleased but I don’t think that’s the strongest motivation – I think the strong motivation is if they feel they're serving humanity, they're helping other people.* [KI 6, Site 1]  *Yeah, I think giving them some incentive is a good idea. I think a voucher that you could spend anywhere may be more beneficial...*[KI 8, Site 1] |
